# Male papillomavirus infection and genotyping in the Qingyuan area

**DOI:** 10.1186/s12985-020-01423-w

**Published:** 2020-10-16

**Authors:** Wei-Guo Yin, Meng Yang, Lei Peng, Yan-Mei Liu, Bin Cheng, Shu-Xia Xuan, Chen Chen, Feng-Jun Tan

**Affiliations:** 1 Department of Molecular Diagnosis Center, The Sixth Affiliated Hospital of Guangzhou Medical University, Qingyuan People’s Hospital, No. B24, Xincheng Yinquan Road, Qingcheng District, Qingyuan, 511518 Guangdong China; 2 Otorhinolaryngology, The Sixth Affiliated Hospital of Guangzhou Medical University, Qingyuan People’s Hospital, Guangdong, 511518 Qingyuan China

**Keywords:** Human papillomavirus, Genotype, Men, Qingyuan area, HPV infection

## Abstract

**Background:**

This study aims to screen the male human papillomavirus (HPV) infection status and genotyping in Qingcheng District, Qingyuan City, Guangdong Province, China to provide a reference basis for formulating prevention strategies for HPV infection.

**Methods:**

The present study collected urethral epithelium or scraped penile epidermis from high-risk male patients in Qingyuan People's Hospital during the last five years, extracted DNA fragments using the boiling method, and detected 23 types of HPV genotypes by PCR-reverse blot hybridization.

**Results:**

The positive detection rate was 54.31% of 1044 males with high risk of HPV (567/1044). Among these males, the positive detection rate of HPV was the highest in patients initially diagnosed with warts, and the rate was 66.47%. Five main HPV types are identified as follows: HPV6 18.87% (197/1044), HPV11 10.25% (107/1044), HPV52 8.81% (92/1044), HPV16 6.90% (72/1044), and HPV51 5.08% (53/1044). Among these HPV-infected patients, single infection mainly by low-risk HPV6 and HPV11 accounted for 56.61% (321/567); high- and low-risk combined HPV co-infections accounted for 29.10% (165/567). The HPV infected patients was mainly between 21 and 40 years old, and the HPV infection rate was higher with increased age.

**Conclusions:**

The HPV infection rate in the Qingyuan area is higher than in other areas and the main infection is single infection. Furthermore, HPV52, HPV16, and HPV51 are the main high-risk infection types, while HPV6 and HPV11 are the main low-risk infection types.

## Background

Human papillomavirus (HPV) is a circular double-strand DNA virus belonging to *Papillomaviridae* family [1]. It has been reported that more than 100 types of HPV have been found. Among these, more than 40 species can infect the genitourinary tract. The International Agency for Research on Cancer (IARC) divides HPV into low-risk and high-risk types according to oncogenic potential risk. High-risk types can be integrated into the host genome. Repeated or persistent infection with high-risk HPV is a necessary condition and main risk factor for cervical cancer in women. The low-risk type is mainly correlated to genital warts [2]. It has been reported that male HPV infection significantly increases the risk of cervical cancer in their sexual partners [3]. Hence, the role of males in the occurrence and development of cervical cancer in females cannot be ignored.

Currently, comprehensive studies of HPV infection and genotypes in men are still rare. In order to improve the understanding of the male HPV infection and genotypes, here we screened the male human papillomavirus (HPV) infection status and genotyping in Qingyuan, China.

## Materials and methods

### Objective of research

This study was a retrospective trial. From January 2014 to September 2018, male patients with high-risk for HPV infection who were hospitalized and received a physical examination in Qingyuan People's Hospital were recruited in this study. These participants were hospitalized due to their disease. HPV infection can be divided into low-risk type and high-risk type according to the pathogenicity or carcinogenic risk. High-risk populations mainly include the following: (1) subjects with symptoms of urethritis, wrapping balanitis, etc.; (2) subjects with suspected condyloma acuminata, papules, and ulcers; and (3) subjects who are conscious of the symptoms of discomfort or have a physical examination. This study was conducted in accordance with the declaration of Helsinki and approved by the Ethics Committee of the Sixth Affiliated Hospital of Guangzhou Medical University and Qingyuan People’s Hospital. Informed consent was obtained from the patient or his guardian.

### Inclusion and exclusion criteria

Inclusion criteria: (1) male patients; (2) patients over 14 years old; (3) high-risk populations for HPV infection. Exclusion criteria: (1) patients with incomplete clinical data; (2) patients with non-conforming specimen collection.

### Sample collection

Urethral epithelium or scraped penile epidermis were collected. Urethral swab was used to sample the urethral epithelium [4]. The swab was inserted ~ 2 cm into the urethra and rotated 360 degrees while removing it. Subjects with verrucous vegetations were scraped with aseptic blades.Samples were placed in separate tubes containing Eagle Minimum Essential Medium, and immediately sent for examination. Specimens that could not be sent in time for examination were stored in a refrigerator at 4 °C or − 20 °C. All specimens were taken from patients in the hospital, and the hospital arranged special personnel to transport them, without any commercial organization to assist in transportation.

### Agents and instruments

The HPV-DNA genotyping (23 types) detection reagent kit was purchased from Shenzhen Yaneng Biological Technology Co., Ltd, which includes 18 high-risk types (HPV16, 18, 31, 33, 35, 39, 45, 51, 52, 53, 56, 58, 59, 66, 68, 73, 83, and MM4) and five low-risk types (HPV6, 11, 42, 43, 81, and 82). The main inspection instruments included the following: Life express PCR expander, which was purchased from Hangzhou Bioer Technology Co. Ltd.; YN-H18 semi-automatic nucleic acid molecular hybridometer, which was purchased from Yaneng BioSciences (Shenzhen).

### DNA extraction

One ml of sample containing medium was transferred to a 1.5-ml centrifuge tube and centrifuged for 10 min at a speed of 13,000 rpm. The liquid supernatant was discarded to retain the cell precipitate at the bottom of the tube. Then, 50 μl of lytic solution was added to the suspended precipitate, centrifuged for 10 min at 13,000 rpm, and heated for 10 min to retain the liquid supernatant containing DNA sample for future use.

### PCR amplification and hybridization

Five μl of the extracted DNA was taken for the PCR reaction under the following conditions: 50 °C for 15 min; 95 °C for 10 min; 94 °C for 10 s, 42 °C for 90 s, 72 °C for 30 s, 10 cycles; 94 °C for 10 s, 46 °C for 60 s, 72 °C for 20 s, 30 cycles; 72 °C for five minutes. After amplification, the membrane strip was marked with the patient number and 2 mL of liquid A and all the corresponding PCR products were lightly shaken and hybridized at 51 °C for 30 min. Liquid A was discarded, 2 ml of liquid B was added and shaken gently for 10 min. Liquid B was then discarded, an incubation solution was prepared according to liquid A:POD = 2000:1, 2 ml of incubation solution was added to each film strip and it was gently soaked at room temperature for 15 min. The incubation liquid was discarded, and each film strip was washed at room temperature with liquid A and liquid C. The film was soaked in 2 ml color-developing liquid for 15 min. Then, 2 ml of deionized water was added into the discoloration solution, it was shaken gently for 3 min, and the results were observed. Result interpretation: (1) the IC site on the membrane strip had a signal interpreted as genotype positive in the corresponding position; (2) blue spots were found at one or more HPV genotypic sites, which were judged to be positive for the genotypic markers at the corresponding position; (3) there was no signal at the IC site on the membrane strip, or at the HPV genotyping site. The human ACTB (β-actin) gene was used as an internal reference. The positive control was the HPV-16 type plasmid, while the negative control was H_2_O. The controls (both internal reference β-globin and HPV-plasmids) were included in the kit. The testing reagent was provided by Shenzhen Yaneng biological company.

### Statistical analysis

In the present study, EXCEL 2007 was used to collate and draw the charts, SPSS 17.0 statistical software was used to process the data, and the measurement data were expressed as mean ± standard deviation. The enumeration data were expressed as percentages (%). It was considered that *P* < 0.05 means that the difference was statistically significant.

## Results

### The relationship of HPV infection and age

In the present study, a total of 1044 male patients were involved in the detection of HPV genotyping. The age of these subjects ranged from 15 years old to 83 years old and the infection rate of HPV in each age group was above 50%. The overall trend of the HPV infection rate increased with age. Among these positive infections, the majority of infected subjects were within 21–40 years old, accounting for more than 70% of the infected population. The specific results are presented in Table 1.

**Table 1:** Age distribution and constituent ratio of HPV infected subjects

Age	n	Positive case (n)	HPV detection rate (%)	Constituent ratio (%)
≦ 20 (years)	53	29	54.72	5.11
21–30 (years)	442	243	54.98	42.86
31–40 (years)	350	186	53.14	32.80
41–50 (years)	147	76	51.70	13.40
51–60 (years)	43	26	60.46	4.59
≧ 61 (years)	9	7	77.78	1.23

*HPV* human papillomavirus

### HPV infection rate

Among the 1044 male high-risk subjects, 567 infected subjects were detected and the infection rate was 54.31%. The HPV infection rate for high-risk types, low-risk types, and high- and low-risk types co-infections was 32.09%, 38.80%, and 29.10% respectively.

### Distribution of subtypes of HPV-infected subjects

There were 23 genotypes in the 567 HPV-infected subjects. Among these, HPV52 was the most high-risk subtype with the highest detection rate (8.81%), and this was followed by the HPV16 (6.90%), HPV51 (5.08%), HPV68 (4.12%), and HPV58 (3.26%) subtypes. The low-risk subtypes with high detection rates were HPV6 (18.87%), HPV11 (10.25%), HPV42 (5.36%), and HPV43 (4.78%) (Table 2).

**Table 2:** Detection rate of the 23 types of HPV in the male high risk population

Genotypes	Positive case (n)	Detection rate (%)
High-risk subtype	468	44.83
HPV16	72	6.90
HPV18	24	2.30
HPV31	10	0.96
HPV33	17	1.63
HPV35	10	0.96
HPV39	22	2.11
HPV45	5	0.48
HPV51	53	5.08
HPV52	92	8.81
HPV53	32	3.07
HPV56	12	1.15
HPV58	34	3.26
HPV59	19	1.82
HPV66	15	1.44
HPV68	43	4.12
HPV73	6	0.57
HPV82	2	0.19
Low-risk subtype	458	43.87
HPV6	197	18.87
HPV11	107	10.25
HPV42	56	5.36
HPV43	50	4.79
HPV81	44	4.22
HPV83	4	0.38

*HPV* human papillomavirus

### Distribution of multiple HPV infections

Among the 567 HPV-infected subjects, the constituent ratio for single HPV infection was 56.61%, which were mainly low-risk HPV6 and HPV11, followed by HPV52, HPV16, HPV42, HPV51, etc. The ratio for double HPV co-infection was 26.63%, and the ratio for high- and low-risk mixed co-infection was 16.40%. The ratio for high- and low-risk mixed infection in multiple infections was 12.70%, and the ratio for low-risk co-infection subjects was very low, only 0.88% (Table 3).

**Table 3:** Distribution of single infection and multiple infection in the HPV subtypes

Type	Positive case (n)	Constituent ratio (%)
Single infection	321	56.61
Single high-risk	131	23.10
Single low-risk	190	33.51
Double infection	151	26.63
Double high-risk	33	5.82
Double low-risk	25	4.41
Double high- and low-risk	93	16.40
Multiple infection	95	16.75
Multiple high-risk	18	3.17
Multiple low-risk	5	0.88
Multiple high- and low-risk	72	12.70

*HPV* human papillomavirus

151 people were infected with 2 types of HPV. 59 patients were screened by physical examination, among whom 25 (43.86%) were aged 21–30 years. HPV6, HP81 and HP11 were the most common types, with 20 patients (35.09%), 9 patients (15.79%) and 8 patients (14.04%), respectively. 54 patients were diagnosed with warts, 21 of them were the ages of 31–40 years (38.89%). HPV6, HPV11 and HPV16 were the three common types, account for 25 patients (46.3%), 15 patients (27.78%) and 8 (14.81%) patients, respectively. Compared with the physical examination population, patients with clinical diagnosis of warts had higher detection frequency of HPV16. The other diagnostic types included foreskin glanthitis, skin infection, dermatitis, urethritis in a total of 38 people (Table 4).

**Table 4:** The ratio and relationship of HPV co-infection types

Coninfection type	Diagnosis type	Cases	Ratio (%)	Common types in co-infection	Cases	Ratio (%)
2-types (151 case)	Physical examination	59	39.07	HPV6	20	35.09
				HPV11	8	14.04
				HPV81	9	15.79
				HPV6 and HPV81	2	3.51
				Other	24	42.11
	Wart			HPV6	25	46.30
				HPV11	15	27.78
				HPV16	8	14.81
				HPV6 and HPV11	2	3.70
				HPV6 and HPV16	2	3.70
				HPV11 and HPV16	4	7.41
				Other	14	25.93
	Other	38	25.17			
≧3-types (95 cases)	Wart	46	48.42	HPV6	25	54.35
				HPV11	16	34.78
				HPV6 and HPV11	7	15.22
				other	12	26.09
	Physical examination	26	27.37	HPV6	9	39.13
				HPV52	14	60.87
				HPV6 and HPV52	5	21.74
				other	8	34.78
	Other	23	24.21			

There were 95 people with three or more types of HPV infection. About half of them (46 patients) were clinically diagnosed with warts, of whom 28 patients (60.87%) were aged 21–30 years. Among them, 25 cases (54.35%) contained HPV6, 16 cases (34.78%) contained HPV11, and 7 cases (15.22%) combined with HPV6 and HP11. Physical examination screened 26 patients with 3 or mor types of HPV co-infections. Nine of them were aged 21–30 years, accounting for 39.13%. There were 14 patients (60.87%) contained HPV52 and 9 patients (39.13%) with HPV6. The remaining 23 cases were clinically diagnosed with skin infection, urethritis, paraphernalitis, skin rash, prostatitis and other related diseases, accounting for 24.21% (Table 4).

### Clinical manifestations of study participants

In this study, we found that the clinical signs of HPV infection included warts, dermatitis, acroposthitis, urethritis, rash, and prostatitis. Warts are skin-surface vegetations caused by the human papillomavirus. Dermatitis is a general term for inflammatory skin disorders caused by a variety of internal, external, or non-infectious factors. Acroposthitis is the inflammation of the foreskin’s inside surface and the penis head. A rash is a skin lesion that can take many forms, from simple skin-color changes to skin-surface bulges or blisters. Urethritis is a common disease that refers to inflammation of the urethral mucosa. Prostatitis (prostatitis) refers to prostate disease caused by a variety of complex causes, with urethral stimulation symptoms and chronic pelvic pain as the main clinical manifestations. The positive detection rate of patients with clinical manifestations was the highest, reaching 66.47% (Table 5).

**Table 5:** Detection rate of HPV in patients with different clinical symptoms

Diagnostic classification	n	Positive case (n)	Positive rate (%)	Constituent ratio (%)
Wart	340	226	66.47	39.86
Dermatitis	117	52	44.44	9.17
Acroposthitis	48	24	50.00	4.23
Urethritis	36	19	52.78	3.35
Rash	33	9	27.27	1.59
Prostatitis	23	7	30.43	1.23
Others	51	25	49.02	4.41

Wart was defined as skin surface vegetations caused by the human papillomavirus

*HPV* human papillomavirus

## Discussion

HPV is closely correlated to a variety of diseases and is one of the more frequent DNA oncoviruses at present. Furthermore, 5.5% of cancers worldwide are associated with HPV infection [5]. HPV infection is commonly detected in clinics and is one of the most common sexually transmitted diseases, especially in the skin and anal genitalia. Considering the severity of HPV, the rapid and accurate detection of HPV infection through genotyping is of great significance for the prevention and treatment of HPV-related diseases. These amplified products were crossed with 17 types of high-risk type and six types of low-risk typing probes on the fixed membrane strip by PCR-reverse blot hybridization. A total of 23 kinds of HPV subtypes common in clinic can be typed simultaneously. This method has the advantages of high accuracy, strong specificity, and high sensitivity. This has been widely used in the typing detection of HPV infection [6].

HPV was epitheliophilic and preferentially infects squamous epithelium rather than columnar, cubed, or transitional epithelium. Therefore, the head of the penis, the body of the penis and the scrotum, the urethral orifice were common sites of HPV infection. A previous study by Ni et al*.* [7] identified 43.7% HPV infection rate in the tumor tissues of male patients with head and neck squamous cell carcinoma. Chen et al. [8] reported that the male HPV infection rate in a clinic in Shanghai was 47.8% by sampling scrotum or penis. In addition, Luo et al. [9] reported that the infection rate of male HPV in the Guangdong area was 57.8%. In this paper, the HPV-typing of urethral epithelium or scraped penile epidermis from 1044 male outpatients in Qingyuan District was carried out and the total infection rate was 54.31% under our sampling methods.

Among the 567 HPV-infected subjects, the single HPV-type infection was dominant (56.61%) and significantly higher than that of the double infection (26.63%) and multiple infection (16.75%). HPV6 and HPV11 were most common HPV subtypes.Although HPV6 and/or HPV11 are mostly latent infections in the genitalia and usually do not show clinical symptoms, these are the dominant genotypes that cause condyloma acuminata in countries around the world [10–13]. The high-risk subtypes of HPV play an important role in the development of penis cancer [11]. Infection with multiple HPV subtypes would increase the risk of abnormal proliferation and canceration of infected cells [14–16]. Therefore, it is necessary to reduce the risk of carcinogenesis of infected tissues by increasing the frequency of follow-ups, performing regular pathological examinations for pathological tissues, or strengthening the treatment for high-risk HPV-infected subjects and multiple HPV subtypes (including high-risk subtypes) [17].

In the present study, it was found that the positive rate of HPV in vegetation was the highest, followed by prepuce glans and uremitis. It was reported that the initial clinical manifestations of HPV virus in Chinese men are often condyloma acuminata, which is vegetations on the genital surface. Condyloma acuminata are most common in the genitals and anus. The color and size of skin lesions vary according to the course of disease [18]. At first, it presents as small, reddish papules. Then, the size gradually expands, the number increases, and the color gradually changes to grayish white or brown-black. The incubation period varies from two weeks to eight months, with an average incubation period of 2–3 months. There are three routes of infection: sexual contact infection, indirect contact infection, and mother-to-child vertical infection [19]. In order to cut the routes of transmission, controlling the source of HPV infection is the key step. Only foreign vaccines have been approved for use in China. Preventive vaccines produced in China have not yet been listed, and phase III clinical trials are still under way. HPV patients also need secondary prevention, and there is no specific method to cure HPV virus. At present the most important mode of treatment is to remove the local hyperplastic warts, which is effective, simple, safe, and scar-free [20]. HPV can also be treated by physical therapy, medicine, and vaccine. In China, in addition to a healthy lifestyle and vaccination, the prevention of HPV infection is performed mainly through uterine screening for primary prevention [21]. However, whether this would be applicable to men and cover the most prevalent genotypes remains unknown and requires further research in the future.

The infection rate of HPV in male patients with venereal disease in the Qingyuan District was high and mainly consisted of low-risk HPV or single-type HPV infections. Patients who were positive for HPV were mainly in the age group of 21–50 years old, and the detection rate was higher in the age group of over 50 years old. HPV52, HPV16, and HPV51 were the three main high-risk HPV types in the region. HPV6, HPV11, and HPV42 were the three main low-risk HPV types in the region. Since there are regional differences in the distribution of male HPV infection types [22, 23], mastering the HPV infection of male patients in local sexually transmitted disease (STD) clinics plays an important role in the monitoring of diseases caused by HPV. In clinical work, in addition to actively treating skin lesions, such as condyloma acuminate, in HPV-infected subjects, especially those with high-risk and multiple HPV infections, close follow-ups should be conducted for a long period of time. This can not only reduce the incidence of penile cancer but also effectively reduce the risk of condyloma acuminatum or cervical cancer.

There remain limitations in the present study. First, the present study was a retrospective study, and not a randomized controlled trial, and no blinding method was established. Therefore, there is still a risk of bias due to causes such as the differing severity of the patient’s disease, the differing duration of the disease, and the previous use of various drugs. Second, the present study is a single-center clinical study. Hence, there is still a need to carry out multicenter clinical studies.

## Conclusion

HPV infection in the Qingyuan District was higher than in other areas, and single infection was the main infection type. HPV52, HPV16 and HPV51 were the major high-risk infection types, and HPV6 and HPV11 were the major low-risk infection types. Therefore, male patients need to regularly check the reproductive tract to reduce the incidence of genital tumors.

## Data Availability

The datasets generated and/or analysed during the current study are not publicly available due to the lack of an online platform but are available from the corresponding author on reasonable request.

## Abbreviations

HPV: Human papillomavirus
IARC: International Agency for Research on Cancer
STD: Sexually transmitted disease
